# Cloning of the Major Capsid Protein (MCP) of Grouper Iridovirus of Taiwan (TGIV) and Preliminary Evaluation of a Recombinant MCP Vaccine against TGIV

**DOI:** 10.3390/ijms161226118

**Published:** 2015-12-02

**Authors:** Hsin-I Liu, Pinwen Peter Chiou, Hong-Yi Gong, Hsin-Yiu Chou

**Affiliations:** 1 Department of Aquaculture, National Taiwan Ocean University, Keelung 20224, Taiwan; duo.liu@msa.hinet.net (H.-I.L.); ppchiou@mail.ntou.edu.tw (P.P.C.); hygong@mail.ntou.edu.tw (H.-Y.G.); 2 Center of Excellence for the Oceans, National Taiwan Ocean University, No. 2, Pei-Ning Rd., Keelung 20224, Taiwan

**Keywords:** grouper iridovirus of Taiwan (TGIV), major capsid protein (MCP), recombinant vaccine

## Abstract

Fish iridoviruses cause systemic diseases with high mortality in various species of wild and farm-raised fish, resulting in severe economic losses. In 1998, we isolated a new epizootic iridovirus in cultured grouper (*Epinephelus* sp.) in Taiwan, thus named as grouper iridovirus of Taiwan (TGIV). We report here the cloning of TGIV major capsid protein (MCP). Phylogenetic analysis of the iridoviral MCPs confirmed the classification of TGIV into the *Megalocytivirus* genus. Recombinant TGIV MCP and GIV MCP were then generated to produce polyclonal antibodies. Western blot analysis revealed that the two antisera were species-specific, indicating no common epitope shared by the MCPs of the two viruses. We further assayed the potency of a subunit vaccine containing recombinant TGIV MCP. The vaccine effectively protected grouper from TGIV infection. The result demonstrated that MCP is a suitable antigen for anti-TGIV vaccines.

## 1. Introduction

Grouper (*Epinephelus coioides*) is a commercially important culture species in many Asian countries. In Taiwan, the yield of grouper in 2013 reached 26,000 tons and was worth of 6.825 billion NT dollars. However, the production of grouper fry has dropped from 282.48 million tails in 1999 to only 65.14 million tails in 2013 [1]. Viral diseases are a major cause to the decline of grouper fry production. Among the viral pathogens, iridoviruses have been reported to cause severe infection in various stages of the life cycle of grouper.

Iridovirus are icosahedral cytoplasmic DNA virus that can infect invertebrates and poikilothermic vertebrates, for example insects, fishes, amphibians, and reptiles [2,3]. Piscine iridoviruses infect a wide range of fish and are classified into three genera: *Lymphocystivirus*, *Ranavirus* and *Megalocytivirus* [4]. While lymphocystiviruse*s* generally instigate non-fatal, superficial dermal infections, ranaviruses and megalocytiviruses are notoriously known for causing high mortality in many economic fish species. Ranaviruseses that are known to infect marine fish include Singapore grouper iridovirus (SGIV) [5] and grouper iridovirus (GIV) [6]. Megalocytiviruses that infect marine fish include red seabream iridovirus (RSIV) [3,7], sleepy grouper disease iridovirus (GSDIV) [8], and infectious spleen and kidney necrosis virus (ISKNV) [9,10,11]. In addition, we have reported the first outbreak of megalocytivirus in cultured grouper in Taiwan, and named the pathogen grouper iridovirus of Taiwan (TGIV) [12].

TGIV could cause up to 60% mortality in the infected grouper fry. Diseased fish show clinical symptoms including swimming in circles and darkening of the body color as a result of anemia. By electron microscopy, abundant number of icosahedral virus particles, of about 230 ± 10 nm in size, are observed in the spleen of diseased fish [12]. Since its discovery in 1998, TGIV has been threatening the grouper fry culture industry in Taiwan [12].

TGIV is equipped with a major capsid protein (MCP) that is of approximately 50 kDa in mass. The MCP is the predominant structural protein in an iridovirus particle and is estimated to account for up to 45% of all virion proteins in the infected cells [13,14]. Virus structural proteins often serve as a key antigen capable of stimulating potent immune response against the viral infection [15]. Hence, MCP has been considered as an important candidate antigen for vaccine against iridoviral infection [16,17]. However, TGIV MCP has not been cloned and characterized up to this moment. In this study, we report the cloning and characterization of TGIV MCP. Furthermore, we tested the potency of a recombinant MCP subunit vaccine against TGIV infection in grouper. The data showed that the vaccine could provide protection with 86% of relative percent survival (RPS) in the infected grouper.

## 2. Results

### 2.1. Sequence Analysis of TGIV-MCP

The TGIV-MCP gene is 1362 bp in length, encoding a putative 453-amino acid protein with a predicted molecular mass of 49.96 kDa (accession number KT989778). Compared to its counterparts in *Megalocytivirus* genus, TGIV-MCP amino acid sequence is 99.8%, 99.8%, 99.6%, 99.6% and 99.3% identical to the MCPs of orange-spotted grouper iridovirus (OSGIV, no. AAX82316.1), grouper sleepy disease iridovirus (GSDIV, no. AAP37443.1), red seabream iridovirus (RSIV, no. BAK14277.1), rock bream iridovirus (RBIV, no. AAW48183.1), and infectious spleen and kidney necrosis virus (ISKNV, no. ADU25248.1), respectively. Additionally, sequence was 61.9%, 59.6% and 59.6% identical to the homologs of *Ranavirus*: Frog virus 3 (FV3, no. ACP19256.1), Grouper iridovirus (GIV, AAV91066.1), Singapore grouper iridovirus (SGIV, no. YP_164167.1), respectively. The phylogenetic tree established on the amino acid sequences of MCPs in megalocytiviruses and ranaviruses support the classification of TGIV into the *Megalocytivirus* genus (data not shown).

**Figure 1:**
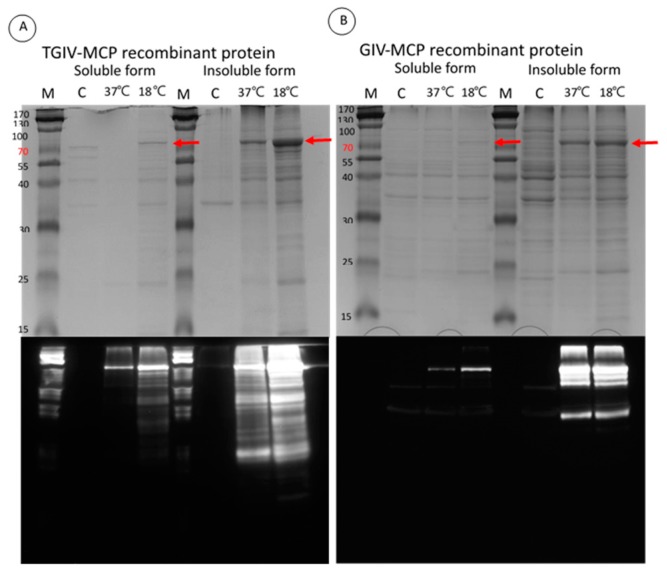
Induction of recombinant TGIV-MCP and GIV-MCP proteins at different temperatures. After addition of IPTG, the transformed *E. coli* cultures (**A**: TGIV-MCP, **B**: GIV-MCP) were incubated at 18 or 37 °C for 12 h. Bacterial cells were harvested and homogenized. Both soluble and insoluble proteins were subjected to SDS-PAGE (**upper** panels), followed by Western blotting with anti-His monoclonal antibody (**A**) or anti-GIV polyclonal antibody (**B**) (**lower** panels). C: sample harvested prior to IPTG induction.

### 2.2. Expression and Purification of Recombinant TGIV-MCP and GIV-MCP for Production of Polyclonal Antibodies

**Figure 2:**
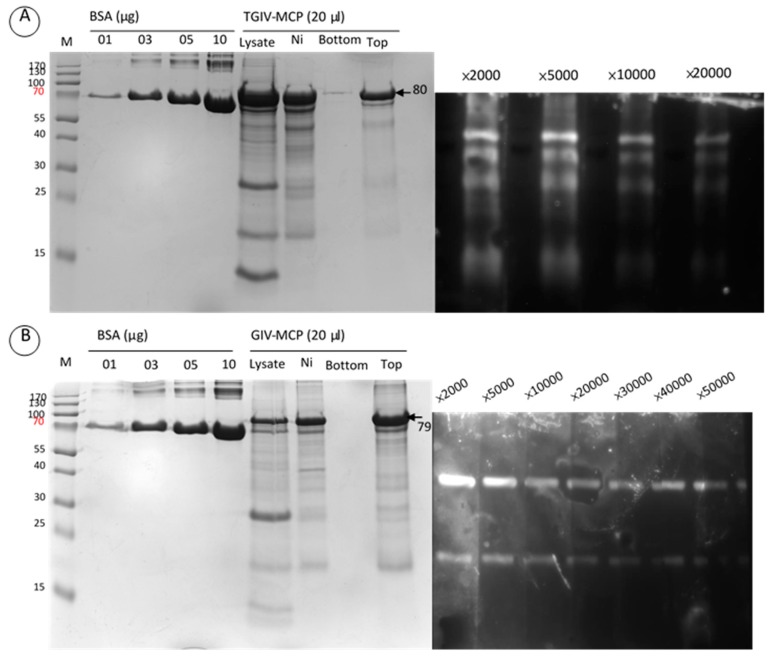
Generation of anti-TGIV–MCP and anti-GIV–MCP polyclonal antibodies. The purification of recombinant MCP proteins and the specificity of the polyclonal antibodies are shown in the left and right panels, respectively. **Left** panels: Recombinant TGIV–MCP (**A**) and GIV–MCP (**B**) proteins were purified by Ni–NTA column (Ni), followed by centrifugation in centricon filter to displace imidazole in the lysis buffer. The purified recombinant proteins were used to immunize rabbit to generate polyclonal antibodies against TGIV–MCP and GIV–MCP, respectively. **Top**: the purified recombinant proteins; **Bottom**: the flow-through waste. **Right** panels: Western blotting was carried out to verify the specificity of the polyclonal antibodies generated from the recombinant proteins. Recombinant TGIV–MCP and GIV–MCP proteins were subjected to SDS-PAGE and transferred to a PVDF membrane, respectively. Western blotting was conducted with anti-TGIV-MCP (**A**) or anti-GIV–MCP (**B**) antisera. The TGIV–MCP antiserum was diluted in PBS at 2000, 5000, 10,000 and 20,000 folds, and the GIV–MCP antiserum was diluted in PBS at 2000, 5000, 10,000, 20,000, 30,000, 40,000 and 50,000 folds.

The pGS-21a-TGIV-MCP prokaryotic expression vector was employed to express recombinant His–GST–TGIV–MCP–His and His–GST–GIV–MCP–His proteins. Optimal expression of both recombinant proteins was achieved by incubation with 1 mM IPTG for 12 h at 18 °C (Figure 1, upper panel). The recombinant proteins were further verified by Western blotting with anti-His monoclonal serum (Figure 1, lower panel) and subsequently purified by Ni–NTA column (Figure 2, left panel). The purified recombinant TGIV–MCP and GIV–MCP proteins were then used to immunize rabbit to generate anti-TGIV–MCP and anti-GIV–MCP polyclonal antibodies, respectively. The avidity of the two polyclonal antibodies was evaluated by Western blotting (Figure 2, right panel). Both antisera could be diluted up to 1:10,000 in the assay.

### 2.3. Specificity of Anti-TGIV-MCP and Anti-GIV-MCP Sera

**Figure 3:**
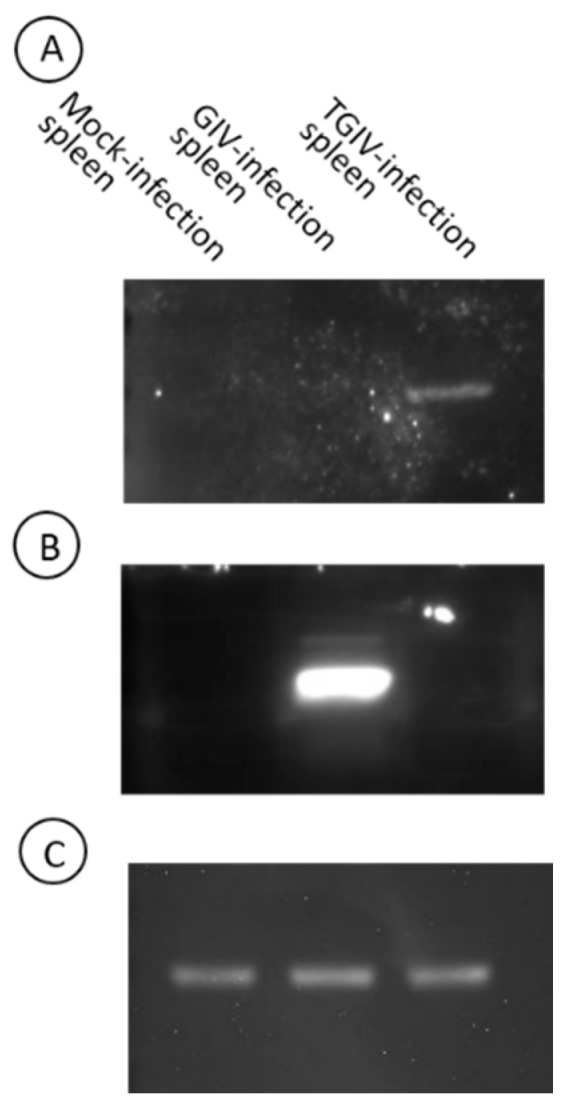
Detection of MCPs in TGIV and GIV infected tissues with specific polyclonal antibodies. The spleen tissues were collected from morbid fish infected with TGIV or GIV. Cell lysates were subjected SDS-PAGE, transferred to a PDVF membrane, and detected by TGIV-MCP (**A**) and GIV MCP (**B**) polyclonal antibodies (5000-fold dilution in PBS), respectively. β-Actin detected with specific monoclonal antibody (**C**) was included as a control.

Grouper fry (0.4 g) were challenged with TGIV and GIV by intraperitoneal injection. Spleen tissue samples were collected at the appearance of typical clinical symptoms and were subjected to Western blot analysis with anti TGIV-MCP and GIV-MCP antibodies. As shown in Figure 3, both TGIV-MCP and GIV-MCP antibodies successfully identified their own target MCP in the infected tissues. Interestingly, there appeared no cross-reactivity between the two antisera as indicated by that each antiserum specifically recognize its own target MCP.

### 2.4. Immunogenicity of Recombinant TGIV-MCP

**Figure 4:**
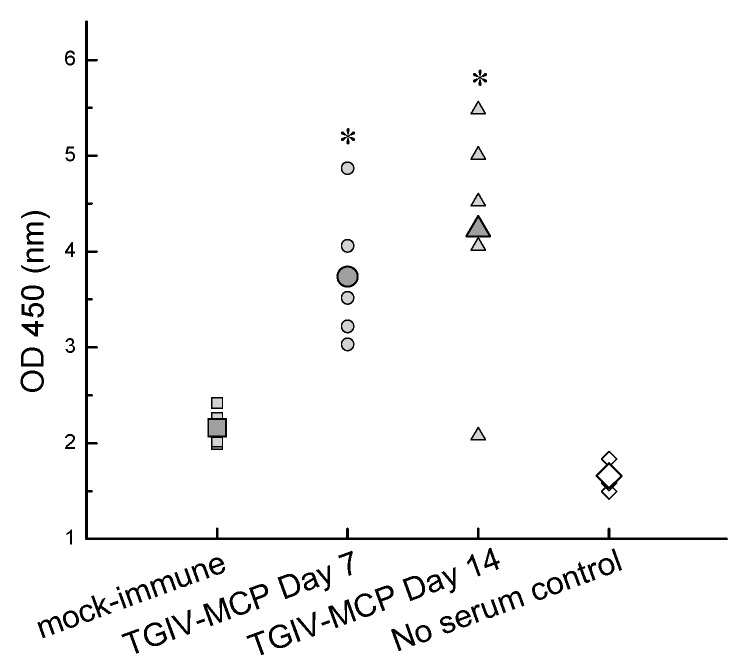
Induction of TGIV-MCP specific antibodies in immunized grouper. Serum samples were taken at 7 and 14 days post-immunization from 5 grouper immunized with recombinant TGIV-MCP protein. The serum samples were assayed by ELISA with anti-TGIV-MCP polyclonal antibodies. The large symbols represent average titer of the five samples in each treatment. * *p* < 0.05.

To verify the immunogenicity of recombinant TGIV-MCP in grouper, sera collected from grouper immunized with the recombinant protein were assessed by ELISA on days 7 and 14 post-immunization. As shown in Figure 4, the titer of TGIV-MCP-specific antibodies was elevated in the immunized individuals as compared with the controls. The result indicates that the recombinant TGIV-MCP protein could be a candidate vaccine against TGIV infection.

### 2.5. Efficacy of Recombinant TGIV-MCP Vaccine

**Figure 5:**
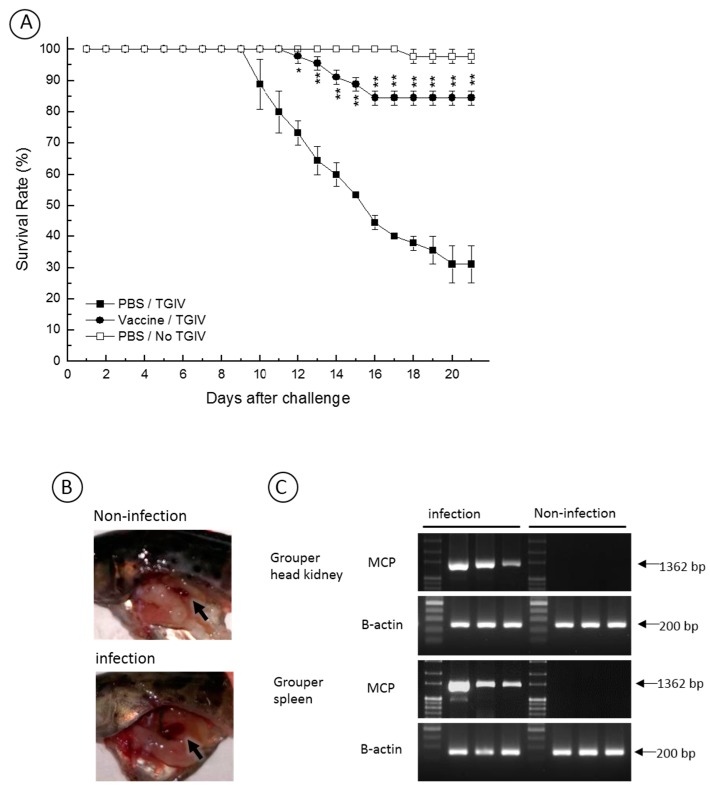
Survival rate of vaccinated and non-vaccinated grouper at different times after challenge with TGIV. (**A**) To test the vaccine efficacy, three treatments were analyzed: (1) vaccination via intraperitoneal injection of recombinant MCP protein, followed by TGIV infection; (2) mock-vaccination via intraperitoneal injection of PBS, followed by TGIV infection; and (3) mock-immunization with PBS only. All groups were carried out in triplicates of 15 fish. The figure shows the average survival rate of the three different groups. The vaccine apparently enhanced the survival rate against TGIV infection with a RPS of about 86% on day 21 post-infection; (**B**) An autopsy was performed on the TGIV-infected and non-infected fish of the mock-vaccinated treatment. Enlargement of spleen, a clinical sign of TGIV infection, was observed in the infected fish but was absent in the non-infected fish. Arrows indicate the spleens; (**C**) The evidence of TGIV replication in the infected fish was assayed. The MCP transcript was detected by RT-PCR in the head kidney and spleen of the infected fish but absent in the non-infected control. * *p* < 0.05–0.01, ** *p* < 0.01.

To evaluate the protection efficacy of the recombinant TGIV-MCP, grouper were immunized via intraperitoneal injection of the recombinant protein, followed by TGIV infection. As shown in Figure 5A, the vaccine enhanced the survival rate in the infected vaccinated fish with a survival rate 84.4% (RPS = 86%) on day 21 post-infection. An autopsy was performed on the TGIV-infected or non-infected control fish. As shown in Figure 5B, enlargement of spleen, a clinical sign of TGIV infection, was observed in the infected fish but was absent in the non-infected fish. We have also investigated the evidence of TGIV replication in the infected fish. As shown in Figure 5C, the MCP transcript was detected by RT-PCR in the head kidney and spleen of the infected fish but absent in the non-infected control. Overall, these data indicate that the mortality was due to TGIV and that the recombinant TGIV-MCP could function as an effective vaccine against TGIV infection in grouper.

## 3. Discussion

Despite the devastating impact of TGIV on the grouper industry, the genome sequence of TGIV remains largely unknown. Being the most predominant structural protein, the iridoviral MCPs have been used to serve as a marker for classification as well as an ideal target for vaccine against the viral infection. Unlike its counterparts in GIV and SGIV of the *Ranavirus* genus, the MCP sequence of TGIV has not yet been determined. We showed here the first report of the nucleotide sequence of TGIV MCP. Further phylogenetic analysis placed TGIV-MCP into the clad of megalocytiviruses. The result provides the first molecular evidence of the phylogenetic position of TGIV in the *Megalocytivirus* genus of the Iridoviridae family.

Antibodies are a powerful tool for virus detection and characterization. For example, Hill and Way have classified aquatic birnavirus infectious pancreatic necrosis (IPNV) into 2 serogroups by serum neutralization assay. Serogroup A contains 9 serotypes, *i.e.*, West Buxton, SP, Ab, He, Te, Canada 1, Canada 2, Canada 3 and Jasper, whereas serogroup B contains only TV-1 serotype [18]. In this study, we have developed polyclonal antibodies against TGIV-MCP and GIV-MCP, respectively. Based on the Western blot analysis, both TGIV-MCP and GIV-MCP effectively interacted with their target protein, respectively. Interestingly, it appeared that there was no cross-reactivity between the two antisera despite a 59.6% similarity in amino sequences between the MCPs of the two viruses. The results suggest that there is likely no common epitope shared by the MCPs of GIV and TGIV. The results further indicate difficulty in developing MCP-based vaccine capable of recognizing both GIV and TGIV. In Taiwan, incidents of GIV and TGIV have been reported annually. Based on the results of this study, future vaccination strategy needs to focus on the development of bi-valent vaccine targeting at both GIV-MCP and TGIV-MCP.

Conventional inactivated vaccines have shown great value in protecting fish from viral infections [19,20]. Successful examples of inactivated vaccines include vaccines for IPNV [21] and Infectious hematopoietic necrosis virus (IHNV) [22] in salmonids, NNV in grouper [23], and RSIV in red seabream [24]. However, the manufacture of inactivated vaccines is labor-intensive and expensive due to the requirement of cultured cells to propagate viruses. Biotechnology vaccines such as subunit vaccines provide an alternate approach. For megalocytiviruses, the viral MCPs have been successfully used to develop subunit vaccine against RSIV, ISKNV and RBIV. Shin and colleagues have developed an oral vaccine containing recombinant RBIV MCP expressed in rice callus [25]. Seo and colleagues have developed another form of RBIV oral vaccine containing recombinant RBIV MCP expressed in yeast [26]. Furthermore, a DNA vaccine targeting at the RSIV MCP has been successfully developed by Caipang and colleagues [27]. Nonetheless, no vaccine against TGIV has been reported so far. In this study, we have developed a subunit vaccine containing recombinant GST-TGIV-MCP fusion protein. The vaccine was capable of assisting grouper against TGIV challenge with a RPS up to 86%. This preliminary test sets the first step to future development of commercial vaccine against TGIV infection in grouper.

## 4. Experimental Section

### 4.1. Cell Culture

Leibovitz’s L-15 medium (Flow Laboratories, Ayrshire, UK) supplemented with fetal bovine serum (FBS) and antibiotics (50 IU/mL penicillin, 50 μg/mL streptomycin, and 1.75 μg/mL fungizone) was used to culture GSB cells derived from grouper swim bladder [28]. For routine passage and virus titration, 10% and 2% FBS was added to the medium, respectively. The cells were incubated at 25 °C.

### 4.2. Grouper

Diseased grouper (2.8 ± 0.5 cm in total length, 0.4 ± 0.15 g in weight) were collected from a culture farm at Kaohsiung Prefecture in southern Taiwan. The healthy grouper were obtained from another hatchery (886 Apex Aquaculture) in southern Taiwan and were verified to be virus-free by polymerase chain reaction (PCR). All of the groupers were maintained in 25–28 °C aquaria with aeration and fed commercially obtained artificial food twice daily.

### 4.3. TGIV Major Capsid Protein Gene Clone

The full-length open reading frame (ORF) of the MCP gene was amplified from infected grouper head kidney and spleen DNA using PCR. TGIV-infected grouper organs were added in a digestion buffer (20 mM NaCl, 200 mM Tris-HCl, 200 mM EDTA, 4 M urea, pH = 8.0) and homogenized by TissueLyser II (Qiagen, Hannover, Germany). The lysate was treated with proteinase K (0.2 mg/mL) at 55 °C for 3 h, and was pelleted down by centrifugation at 2000× *g* for 10 min. The supernatant was subjected to treatment of phenol/chloroform/IAA (25:24:1), and was precipitated with ethyl alcohol. The DNA pellet was dissolved in TE buffer (10 mM Tris-HCl, 1 mM EDTA, pH = 8.0) and used as a template for PCR. The cloning primers, designed based on RSIV-MCP (accession number AB461856), were as follows: MCP-forward: 5’–ATGACTTGTACAACGGGTGC–3’; MCP-reverse: 5’–TTACAGGATAGGGAAGCCTG–3’. The PCR amplification reaction conditions were as follows: 1 cycle of 94 °C for 5 min; 35 cycles of 94 °C for 30 s, 50 °C for 30 s, and 72 °C for 2 min, followed by incubation at 72 °C for 10 min. The TGIV-MCP PCR product was cloned into yT&A vector (Yeastern Biotech, Taipei, Taiwan) for sequencing. The MCP gene sequence was determined by sequencing at least twice. Multiple sequence alignments of TGIV MCP with the counterparts of other iridoviruses were conducted with the program ClustalW. A phylogenetic tree based on the amino acid sequences of iridoviral MCPs was constructed with neighbour-joining method [29] using the MEGA 6.06 program [30].

### 4.4. Expression of Recombinant TGIV Major Capsid Protein

The TGIV MCP ORF was cloned into a pGS-21a *E. coli* expression vector to generate the plasmid pGS-21a-TGIV-MCP. The cloned plasmid was transformed into *E.* *coli* BL21 via heat shock method and cultured in the LB-plated containing ampicillin antibiotic. The pGS-21a-TGIV-MCP expressed the MCP fusion protein with glutathione *S*-transferase protein (GST) and 6-histidine tagged in *E. coli* under the induction of isopropyl-1, 1-thio-β-d-galactopyranoside (IPTG). The expression of the recombinant TGIV MCP was confirmed by sodium dodecyl sulfate polyacrylamide gel electrophoresis (SDS-PAGE). After confirming the size of the recombinant protein, the protein was solubilized with 0.5% sarcosin, and purified by affinity chromatography using a nickel–nitrilotriacetic acid (Ni–NTA) column according to the manufacturer’s instructions (GE)

### 4.5. SDS-PAGE

The quality of recombinant TGIV-MCP protein was analyzed with 10% SDS-PAGE [31]. The protein samples were resuspended in laemmli sample buffer at 95 °C for 5 min, prior to electrophoresis at content current of 100 V. The proteins were visualized by staining with coomassie brilliant blue after electrophoresis.

### 4.6. Western Blot

The identity of the recombinant TGIV-MCP protein was further verified by Western blot. After electrophoresis, the separated proteins on SDS-PAGE were transferred onto a polyvinylidene difluoride (PVDF) membrane. After transfer, the PVDF membrane was soaked in blocking buffer (5% skim milk in PBS-T) at 25 °C for 2 h, followed by incubation with primary antibody for 2 h. The PVDF membrane was washed 5 times in PBS-T (0.2% tween 20 in PBS). Subsequently, the membrane was incubated in solution containing horseradish peroxidase (HRP) conjugated secondary antibody for 1 h, followed by incubation with Immobilon Western Chemiluminescent HRP Substrate (Merck Millipore, Darmstadt, Germany).

### 4.7. Analysis of Antibody Production in Response to Immunization

After 7 and 14 days post-immunization, the levels of anti-TGIV-MCP antibodies in fish from each treatment group were assayed by enzyme-linked immunosorbent assay (ELISA). Fifty nanograms per well of purified recombinant TGIV-MCP protein was coated onto the wells of ELISA plate and let dry in a biosafety cabinet overnight. The plates were washed with PBS, and then blocked with PBS containing 2% BSA at 37 °C for 1 h. Subsequently, the plates were washed with PBS-T three times, followed by incubation with sera from the vaccinated/control grouper at 37 °C for 2 h. The plates were washed with PBS-T three times each for 2 min. After washing, the plates were incubated with 100 μL of anti-grouper IgM monoclonal antibody for 2 h. The plates were then washed with PBS-T for 2 min three times and further incubated with 100 μL of HRP-conjugated anti-mouse IgG for 1 h. After washing with PBS-T for 2 min five times, the plates were incubated with substrate TetraMethylBenzidine (TMB) (Thermo Fisher Scientific, Waltham, MA, USA), and the optical density was measured at 450 nm using an automated ELISA reader (Thermo Fisher Scientific, Waltham, MA, USA).

### 4.8. Vaccination and TGIV Challenge

To evaluate the efficacy of the recombinant MCP protein as vaccine, juveniles of grouper (averaged 2.8 cm in body length and 0.4 g in body weight) were used in the vaccine trials. Groupers were divided into three groups (15 fish per group) for the vaccine trial, which was repeated three times. In group 1, groupers were injected with multiple emulsified PBS (negative control). In group 2, groupers were intraperitoneally injected with 2 μg/fish-g of multiple emulsified recombinant TGIV-MCP. In group 3 (non-vaccinated group), the groupers were intraperitoneally administrated with blank multiple emulsion PBS. After 1 week, booster was carried out with equal dose of recombinant TGIV-MCP. At 2 week post-vaccination, fish from groups 1 and 2 were intraperitoneally injected with TGIV at 1 × 10^4^ copies·fish^−1^. At 21 days post-infection, the survival rate of each group was recorded daily and autopsy was performed to assess the cause of death. Relative percent survival was calculated using the following formula: RPS = [1 − (vaccinated mortality %/control mortality)] × 100% [32]. The significance of difference within treatments was analyzed by Duncan’s multiple range test. For each treatment, 3 repeats were conducted to obtain the mean values, which were considered to be significantly different at *p* < 0.05 [33].

### 4.9. Reverse Transcription-PCR, RT-PCR

Total RNA was extracted from grouper spleen and head kidney by using RNAzol^®^RT (Molecular Research Center, Inc., Cincinnati, OH, USA), according to manufacturer’s recommendations. 1 µg of RNA sample was used for the generation of cDNA by using HiScript I™ Reverse Transcriptase (Bionovas, Toronto, ON, Canada) with final volume of 20 µL according to manufacturer's recommendations.

PCR reaction of TGIV-MCP and β-actin genes was performed using the Taq DNA Polymerase 2× Master Mix (Ampliqon, Odense, Denmark), with final concentrations of 1 µL of cDNA, 1× PCR buffer, 1.5 mM of MgCl_2_, 0.2 mM of dNTP mixture, and 0.2 µM of each primer in a total reaction volume of 25 µL. PCR was performed with an initial incubation at 95 °C for 3 min, followed by 40 amplification cycles (95 °C for 30 s; 54 °C for 30s; 72 °C for 2 min (MCP) or 30s (β-actin)), and a final incubation at 72 °C for 10 min. The PCR products (TGIV-MCP gene: 1362 bp; β-actin: 200 bp) were subjected to electrophoresis in 2% agarose gel, and visualized by staining with ethidium bromide.

## 5. Conclusions

In conclusion, we reported the cloning of TGIV MCP gene. Comparison of the deduced amino sequences of MCPs among ranaviruses and megalocytiviruses confirms the classification of TGIV into *Megalocytivirus* genus. We further demonstrated the potency of a recombinant TGIV MCP vaccine. The results indicate that MCP is an idea candidate antigen for vaccines against TGIV. Lastly, the species-specific TGIV MCP antiserum can be used to differentiate GIV and TGIV infections during disease outbreak.
